# Acute Belladonna Poisoning—Profuse Epistaxis—Recovery

**Published:** 1890-03

**Authors:** William C. Krauss

**Affiliations:** Buffalo, N. Y.; 176 Franklin Street


### Clinical Reports.

### ACUTE BELLADONNA POISONING.—PROFUSE EPISTAXIS.—RECOVERY.

By WILLIAM C. KRAUSS, M. D., Buffalo, N. Y.

On December 15, 1889, I was called to see a patient at Attica, N. Y., who had been ill several days with the mumps. I found the patient to be a woman about 35 years old, strong, robust, with a double parotitis, and temperature of 101° F. I prescribed a light antipyretic, gave the usual advice in such cases, and promised recovery in several days.

On the second day after my visit, a so-styled nurse visited the patient and strongly insisted on her taking belladonna. "Oil of belladonna" was forthwith procured at a neighboring drug store, and, after some manipulation, was administered. After several doses the patient felt her heart beating rapidly, the temples swell, accompanied with strange psychical disturbances, and excessive dryness of the mouth. For a few moments she was in ecstasy, "never felt happier in her life," began to laugh and sing, and tried to leave the bed. Soon afterward her head became exceedingly heavy and dizzy, with painful pulsation at the vertex, perspiration and incoördination followed. An attendant present says her eyes were glassy and had a most peculiar expression, her face was extremely congested, and her delirium seemed to grow continually worse. No physician was summoned. While suddenly turning in bed, her nose began to bleed profusely, *the blood* seeming to leave the nostrils in jets,—something after the manner of projectile vomiting. After three towels had been saturated with blood the hemorrhage ceased, and the patient felt instantaneous relief from all symptoms, save a dull, heavy headache, which persisted several days.

At the end of the week I had occasion to call, and heard the above description, which I considered to be the symptoms of an acute belladonna poisoning. The epistaxis occurring, as it did, at the acme of the attack, and the instantaneous relief it afforded, might suggest the employment of venesection as an antidotal measure in such cases.

176 FRANKLIN STREET.

